# Anti-leishmanial effect of the hydroalcoholic extract of the leaves, roots and seeds of *Arctium lappa*

**DOI:** 10.22038/AJP.2024.24940

**Published:** 2025

**Authors:** Elham Maleki, Afsaneh Yegdaneh, Sakineh Akbari, Saberi Sedigheh

**Affiliations:** 1 *Department of Parasitology and Mycology, School of Medicine, Isfahan University of Medical Sciences, Isfahan, Iran*; 2 *Department of Pharmacognosy, School of Pharmacy and Pharmaceutical Sciences, Pharmaceutical Sciences Research Center, Isfahan University of Medical Sciences, Isfahan, Iran*; 3 *Department of Parasitology and Mycology, School of Medicine, Shiraz University of Medical Sciences, Shiraz, Iran*

**Keywords:** Anti-leishmanial, BALB/c mice, Arctium Lappa, MTT, Glucantime

## Abstract

**Objective:**

Zoonotic cutaneous leishmaniasis is a common including endemic infectious disease in many parts of the world and Iran. Due to *Arctium lappa *wide therapeutic applications, the anti-leishmanial effect of the hydroalcoholic extract of its leaves (L), roots (R) and seeds (S) has been investigated in this research.

**Materials and Methods:**

The leaves, seeds and roots of the greater burdock plant were extracted. In the *in vitro* phase, its cytotoxic and anti-leishmanial effects on promastigote and amastigote forms of* Leishmania major(L.major)* were investigated. In the in vivo stage, the leishmaniasis mouse model was dosed with concentrations of 50, 100, and 200 mg/kg and the liver and spleen parasite burden was checked.

**Results:**

The results of this research in the *in vitro* phase showed that the antileishmanial effect of the hydroalcoholic extract of leaves, roots and seeds on the promastigote and amastigote forms of *L. major* has a significant relationship with the increase in the concentration of the extract (for all p≤0.001). Also, exposure time and interaction effect of concentration and exposure time were significant. In the *in vivo* phase the significant effect of the increase in concentration (L: p≤0.001, R: p=0.02, S: p=0.03), exposure time (L: p≤0.001, R: p≤0.001, S: p≤0.01) and the interaction effect of these two factors (L: p=0.002, R: p≤0.001, S: p≤0.001) on reducing the size of the wound was shown.

**Conclusion:**

The investigation established that hydroalcoholic extract of the leaves, roots, and seeds of the greater burdock in high concentration exhibited beneficial inhibitory effects on the leishmanial lesions.

## Introduction

Leishmaniasis is a zoonotic disease transmitted by a flagellated protozoan called Leishmania from the Trypanosomatidae family. This disease has spread in all parts of the world, including tropical and semi-tropical regions, and in 98 countries throughout Asia, Europe, Africa. According to the report of the World Health Organization, about 1.5 to 2 million people are infected with various types of Leishmania every year, of which 70,000 people die. (Saini et al., 2022; WHO, 2023). This disease is hyper-endemic in some areas of Iran and 95% of cases are related to cutaneous leishmaniasis with an incidence rate of 31 cases per thousand people (Shirzadi et al., 2015). The pentavalent antimonials including glucantime (meglumine antimoniate) and pentostam (sodium stibogluconate), are the main treatments and are administered by intramuscular or intralesional injection. The use of these medicines is associated with side effects including heart, liver and kidney disorders and other difficulties such as importation and scarcity and therefore lack of access for patients in rural areas. Recurrence due to incomplete or insufficient treatment and, the emergence of resistant strains due to mutation and drug resistance has been previously noted (Aronson et al., 2017; Ponte-Sucre et al., 2017). On the other hand, the medicinal properties of florae have always been of interest to researchers and many studies have shown the beneficial effects of their various naturally occurring compounds. For example, the anti-leishmanial effects of plant extracts such as thyme, yarrow, henna and garlic as well as *Ganoderma lucidum* fungi have been investigated to deduce the potential efficacy on the improvement of cutaneous leishmaniasis (Hejazi et al., 2009; Akbari et al., 2019). Also, in other studies, the anti-leishmanial effect of the fruit and flower of Punica granatum plant (Mardani et al., 2020), and the phenolic fractions isolated from plants such as olive, bay leaves and black alfalfa have been mentioned (Gharirvand et al., 2020). 

One herbaceous plant with great therapeutic potential is* Arctium lappa*, or the greater burdock, which has long been associated with unique properties in traditional Chinese medicine. This is an herbaceous plant from the family of chicory and marguerite daisy (Compositae (Asteraceae), grows wild in most parts of the world and has been used as a traditional herbal medicine in Asian as well as western countries for centuries. In Iran, it has been seen in Arak, Tabriz, Mazandaran, Alborz and Gorgan provinces (Boldizsár et al., 2010; Esmaeili et al., 2022). The active compounds described within the plant include flavonoids, arctiopicrin, protein, carbohydrates, inulin, polystyrene compounds, caffeic acid, chlorogenic acid, arctin/arctigenin, cynarin and vitamin C which its potential therapeutic properties on the immune system, anti-malignant effects, antiviral, antibacterial and antifungal, the treatment of skin diseases such as acne and skin pimples is attributed to these compounds (Boldizsár et al., 2010; Sohn et al., 2011). Various biologically active compounds, such as terpenoids (beta-eudesmol, C15H24O, present in the fruit), sterols (sitosterol-beta-d-glucopyranoside, C35H60O6, contained in the root), lignans, polyphenols, and fructans, are found in the plant but each part of the burdock plant has a different composition, especially in terms of bioactive compounds. The roots of burdock contain arctiin, luteolin, quercetin rhamnoside, and it is a remarkable source of proteins, potassium, calcium, folate and is rich in phytochemicals. Burdock possesses antioxidant and antiinflammatory properties and is reportedly pharmacologically active as an anti-diabetic agent, which improves blood lipid profiles, hypoglycemia, and hyperinsulinemia (Ha et al., 2021). In addition to antioxidant properties, other studies have indicated antimicrobial effects (Gram-negative and Gram-positive bacteria) and antiviral activity against herpes virus (HSV-1 and HSV-2), HIV and adenovirus, which can be attributed to the presence of compounds such as caffeic acid, rutin, hydrobenzoic acid, chlorogenic acid and p-coumaric acid in burdock extracts (Parham et al., 2020). Burdock leaves contain chelating metals and elements such as sodium, magnesium, selenium and zinc as well as effective compounds such as arctiol, fukinone, fukinanolide, β-eudesmol, petastilone, eremophilone, taraxasterol, polyphenol, chlorophyll, carotenoid, tannin, arctigenin and glycoside arctin which have a variety of biological activities, such as anti-cancer, anti-HIV, antioxidant and antimicrobial effects. The chemical compounds in the burdock seeds include a bitter glucoside called arctin, chlorogenic acid, lapaul A and B and germacranolide (Jafari Shiran et al., 2022; Mir et al., 2022). Some studies have shown that the different organs of the *Arctium lappa* such as roots, leaves, seeds and fruits may have different therapeutic uses. The roots and seeds are indicated as diuretics and blood cleansing while the leaves are indicated for the treatment of burns, ulcers and skin healing (Ferracane et al., 2010; Luquis et al., 2021). Few studies have investigated the anti-parasitic properties of *Arctium lappa* plant, among which Farghi Yamchi et al study evaluated the anti-leishmanial properties of the methanol extract of plant's root *in vitro* (Farghi Yamchi et al., 2016). Considering the extensive properties of this plant, this study was designed to investigate the anti-leishmanial properties of the leaves, seeds and roots of the native and Iranian species of *Arctium lappa* in vitro and in a laboratory animal model. 

## Materials and Methods

### In vitro stage

#### Preparation of hydroalcoholic extract of leaves, roots and seeds of Arctium lappa 

Burdock leaves, roots and seeds were purchased from a medicinal plant company between May and July 2021 and were approved by the Pharmacognosy Department of the Faculty of Pharmacy and Medicinal Plants, Isfahan University of Medical Sciences, with the herbarium code 2084. Hydroalcoholic extracts were prepared using the maceration method and 64.5 g of seeds, 345 g of roots and 539 g of plant leaves were mixed with 1000 ml of 70% ethanol separately. After 48 hr, the mixture was filtered using Whatman paper and a vacuum and subsequently underwent distillation. In this way, the yields of extraction for leaves, roots and seeds of the plant were 3.7%, 2.9% and 7.6%, respectively. 

### Cultivation of Promastigote

The standard strain of Leishmania parasite (MRHO/IR/75/IR) was provided by the Department of Parasitology, Faculty of Medicine, Isfahan University of Medical Sciences. The parasite was cultured in NNN medium (Novy Mac Neal-Nicolle) and subsequently transferred to RPMI-1640 medium (Roswell Park Memorial Institute) enriched with fetal bovine serum (FBS 10%) and antibiotics (streptomycin 100 mg/ml and penicillin 100 unit/ml).

### Examining the effect of the extracts on promastigote form by MTT method

In this part, 100 µl of culture medium containing 1×10^5^ promastigotes in logarithmic phase was added to the wells of a 96-well plate. Then, 100 µl of the extracts of leaves(L), roots(R) and seeds(S) of burdock plant in concentrations of 12.5, 25, 50, 100, 200, and 400 µg/ml were added to each well (n=2). The positive control wells contained Glucantime as well as amphotericin B and the negative control contained only parasite promastigotes. In order to control the effect of the solvent of the extracts, which was dimethyl sulfoxide (DMSO), and also for the blank (culture medium without promastigotes and active compounds), two wells were considered for each.The plates were incubated at 24°C for 24 and 48 hr. Then 20 µl of 5% MTT (3-(4,5-dimethylthiazol-2-yl)-2,5-diphenyl tetrazolium bromide) solution was added to each well and placed in aluminum foil in an incubator for 4 hr. In the next step, 100 µl of DMSO was added to each well and incubated for 15 min. Then, the optical absorption of the plates was read at a wavelength of 570 nm (Neubig et al., 2003). The survival percentage was calculated using the following formula:

(AT-AB) / (AC-AB) × 100) = survival percentage of promastigotes (%)

AT: optical absorption of treated cells

AC: optical absorbance of untreated cells

AB: Blank optical absorption:

Determination of half-maximal inhibitory concentration (IC50) was carried out using nonlinear regression based on the following formula (Oliveira et al. 2018): 

Log (IC_50_) = log(x1) + [y1-y0/2)/ (y1-y2)] [log(x2)-log(x1)] 

### Investigating the effect of extracts on the promastigote form by counting with a hemocytometer

In this part, 100 µl culture medium containing 77×10^4^ parasites in logarithmic phase were transferred to the wells of 96-well plates. Then 100 µl of six concentrations of 12.5, 25, 50, 100, 200, and 400 µg/ml of the extract of leaves, roots and seeds of the plant was added and after 24 and 48 hr, the number of live and mobile parasites was counted and these steps were repeated twice. Two wells with promastigote without extract were utilized as negative control, whereas Glucantime (50 μg/ml) as well as amphotericin B (0.6 μg/ml) was applied as a positive control. The samples were counted using a hemocytometer and to calculate the number of viable cells/mL, the following formula was used:

The average cell count in 5 RBC chamber × 10^4 ^× 25 × dilution factor 

### Preparing the amastigote-macrophage model and investigating the effect of the extracts on the intracellular form of the parasit

Since macrophage is the best cell for creating the amastigote form of the parasite and investigating the effect of compounds or drugs on it, and usually J774 murine macrophage cell line is used in many researches therefore in this research J774A1 cell line was purchased from Tehran Pasteur Institute and used to preparation the amastigote-macrophage model (Santiago et al., 2022; Sherafati et al., 2023). After the growth of macrophages, they were separated from the bottom of the flask using a cell scraper, washed, centrifuged and counted. A cell count of 15×10^4^ macrophage /ml enriched RPMI 1640 (20% FBS and antibiotics penicillin and streptomycin with concentrations of 100 IU/ml and 100 μg/ml, respectively) was poured into the each well of the 6-well plate, whose bottoms were previously covered with a sterile slide (22×22mm) and placed in an incubator with conditions of 37^o^C and 5% CO_2_. After 24 hr and the adhesion of macrophages to the lamellae, 250 μl containing 15×10^5^metacyclic promastigotes (10 parasites / macrophage) were added to the wells and transferred to the incubator again. After 6 hr, the wells were washed with sterile phosphate-buffered saline PBS, and fresh culture medium was added to the plates. After 24 hr, concentrations of 12.5, 25, 50, 100, 200, and 400 µg/ml of each of leaves, root and seed extracts were added to the wells. Plates without any treatment served as negative control whereas plates administered with Glucantime as the positive control. After 24 and 48 hr, the sterile coverslips that were placed in the bottom of the plate were removed and, first fixed with methanol and subsequently stained with Giemsa dye. The number of amastigotes was determined per 100 macrophage using light microscopy and IC_50_ values were determined. 

### Determination of cytotoxicity of extracts by MTT method

In this part, 100 µl of 5×10^4^ macrophages was transferred to a 96-well plate. Then, 24 hr later, 100 µl of the extracts prepared with concentrations of 12.5, 25, 50, 100, 200, and 400 µg/ml was added. After 24 and 48 hr, the survival of macrophages was calculated using the MTT method mentioned above and CC_50_ values were determined. 

### In vivo phase

#### Investigation of the effect of prepared extracts on the animal model of cutaneous leishmaniasis

At this stage, 5, 10 and 20% topical creams were prepared using 2% carbomer gel (Saeedi et al., 2018). To prepare an animal model of cutaneous leishmaniasis, 5-6 week old female BALB/c mice were used, and 0.1 ml containing 1×10^6^ metacyclic promastigote prepared in the previous part was injected intradermally at the base of the tail by insulin syringe. After four weeks, cutaneous leishmaniasis lesions appeared at the injection site. A total of 55 BALB/c mice were separated into 11 test groups consisting of 5 mice each, in which 9 groups were treated with hydroalcoholic extracts of leaves, roots, and seeds with concentrations of 50, 100, and 200 mg/kg. The 10th group treated with Glucantime and the 11th group without any treatment were the positive and negative controls respectively. The treatment with the extracts was done twice a day with a time interval of 10-12 hr and using a sterile swab for up to 4 weeks. Before and at the end of each week of treatment, the wounds were measured and recorded using vernier calliper, and the percentage of wound reduction was calculated using the following formula:

Percentage reduction in wound size = (The size of the wound on day 0 - the size of the wound on the day n)/ The size of the wound on day 0 × 100.

Finally, the liver and spleen of the infected mice were removed and the parasite load was calculated using the following formula (Grant et al., 2004):

 (body weight in grams) × number of amastigotes per 2000 nucleated cell

### Statistical analysis

Statistical analysis was done by SPSS 20 software. IC50 and CC50 were calculated from the nonlinear regression curve from the log of the inhibitor concentration versus the normalized response. Also selectivity index (SI) which is a ratio CC50:IC50 were calculated. All data was first checked for normality by the Kolmogorov-Smirnov test. The data were compared among experimental groups using Two-way ANOVA and Tukey’s post hoc and a p˂0.05 was considered to be statistically significant. All analyses were performed with GraphPad Prism 6 sofware (GraphPad Sofware Inc.).

## Results

**Figure 1:**
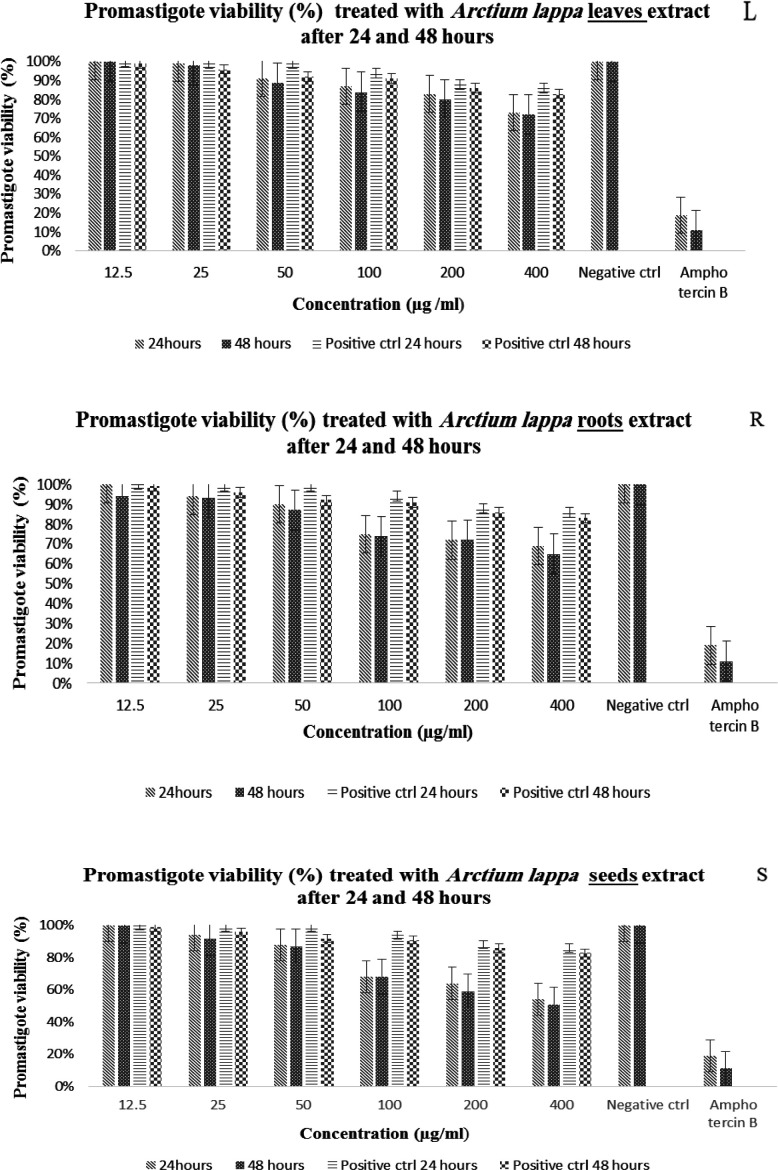
The viability% of promastigotes after exposure to different concentrations of leaves, roots and seeds of *Arctium* *lappa* after 24 and 48 hours using MTT method. L,leaves; R, roots; S,seeds. Data analysis with two-way ANOVA method showed the significant effect of the concentration of each extract (L: p≤0.001 R: p≤0.001 (S: p≤0.001), exposure time (L: p=0.002, R: p=0.004, S: p≤0.001(and the interaction effect of these two factors (L: p≤0.001, R: p≤0.001, S: p≤0.001) on the viability of promastigote form. The results illustrated that the highest inhibitory effect on promastigotes of *L. majar* is respectively related to the seed (IC50 = 480 and 420 µg/ml) then the root ((IC50 = 983 and 771 µg/ml) and finally the leaf (IC50 = 1025 and 920 µg/ml). Positive control (Positive ctrl), Negative control (Negative ctrl).

**Figure 2:**
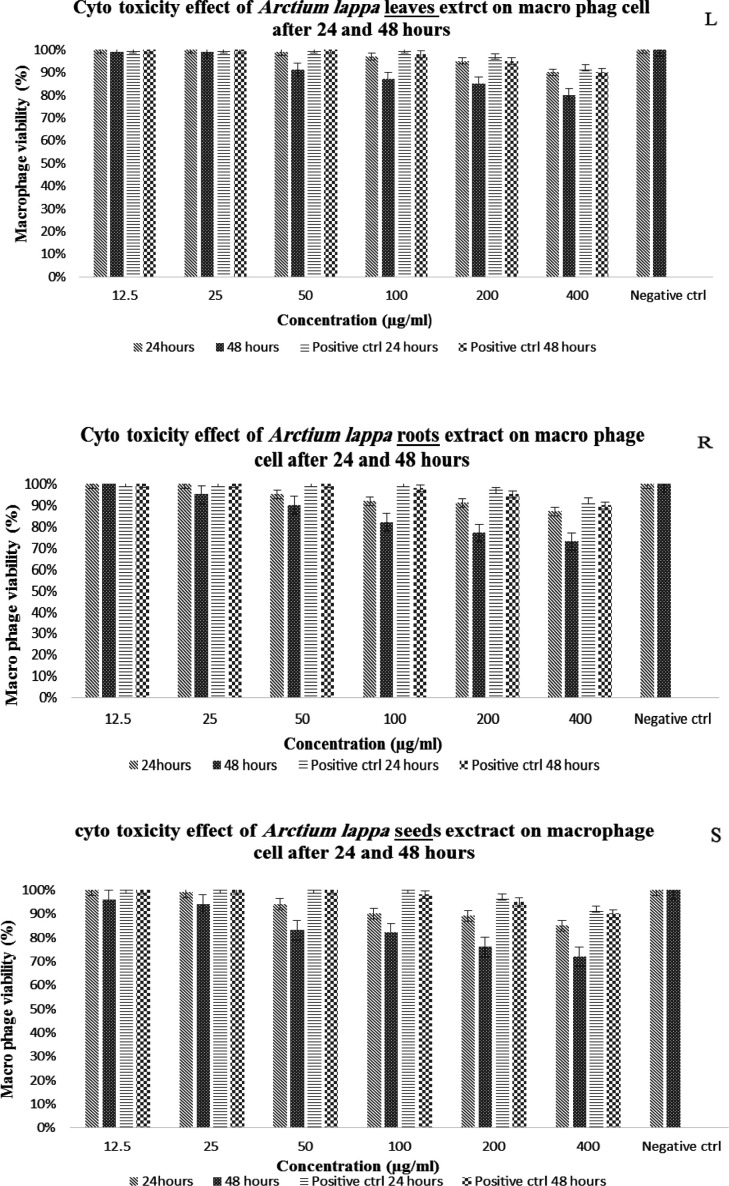
The viability (%) of macrophage cells after exposure to different concentrations of leaves, roots and seeds of *Arctium* l*appa* after 24 and 48 hours. L,leaves; R, roots; S,seeds. The results of the data analysis by two-way ANOVA statistical tests showed the significant effect of the concentration of the extracts (L: p≤0.001, R: p≤0.001, S: p≤0.001), the exposure time (L: p≤0.001, R: p=0.004, S: p≤0.001) and the interaction effect of these two factors (L: p=0.002, R: p=0.019, S: p=0.024) on the viability of macrophages. Also, by performing Tukey's post hoc test, it was found that there is a significant relationship between the different concentrations of the hydroalcoholic extract of the leaves, roots and seeds of the *Arctium* *lappa* with each other and with the negative control (p<0.05). In the period of 48 hours, there was a significant difference between high concentrations (100, 200, and 400 µg/ml) and the positive control (p<0.05). Positive control (Positive ctrl), Negative control (Negative ctrl).

**Figure 3:**
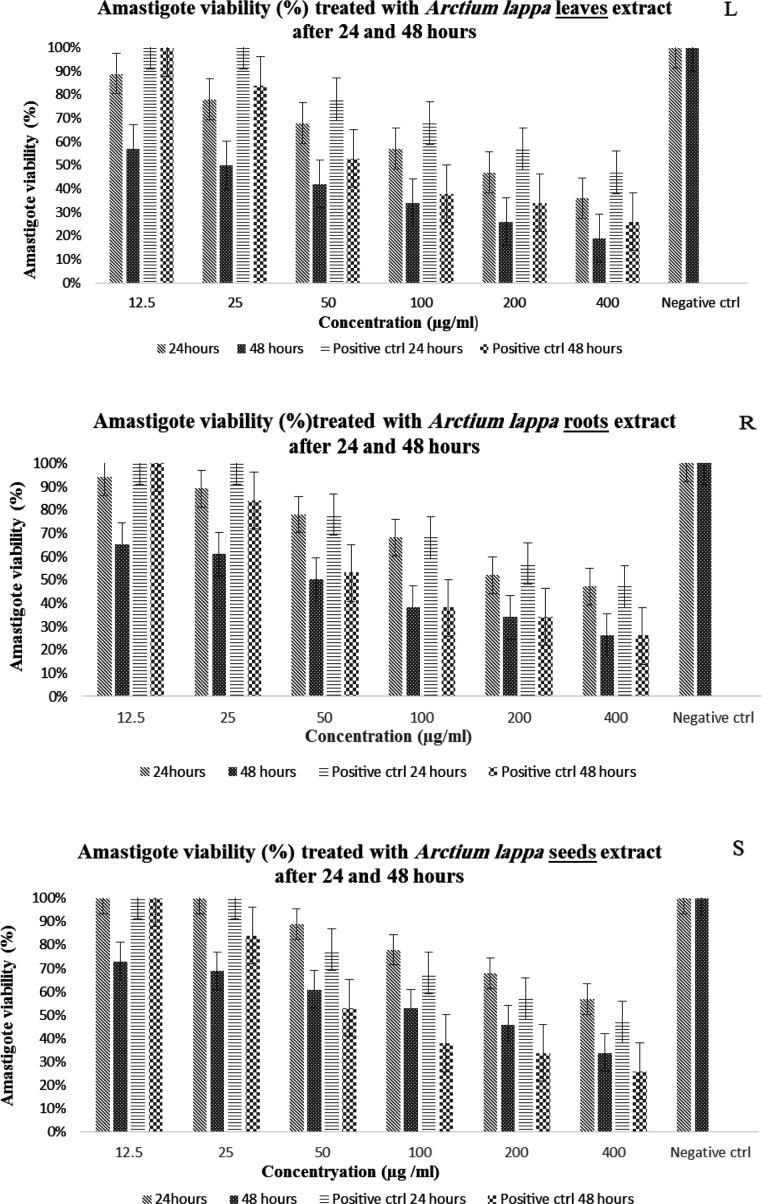
Viability % of amastigotes after exposure to different concentrations of the three types of extracts in 24 and 48 hr. L,leaves; R, roots; S,seeds. Data analysis with two-way ANOVA method showed the significant effect of the increasing concentration (L: p≤0.001, R: p≤0.001, S: p≤0.001), increasing contact time (L: p≤0.001, R: p≤0.001, S: p≤0.01) and the interaction effect of concentration and exposure time (L: p=0.002, R: p≤0.001, S: p≤0.001) on reducing the intracellular form of the parasite. Also, by performing Tukey's post-hoc test, it was found that there was a significant relationship between different concentrations of hydroalcoholic extract of leaves, roots and seeds of *Arctium* *lappa* and the positive and negative controls (p<0.05). Positive control (Positive ctrl), Negative control (Negative ctrl).

**Figure 4:**
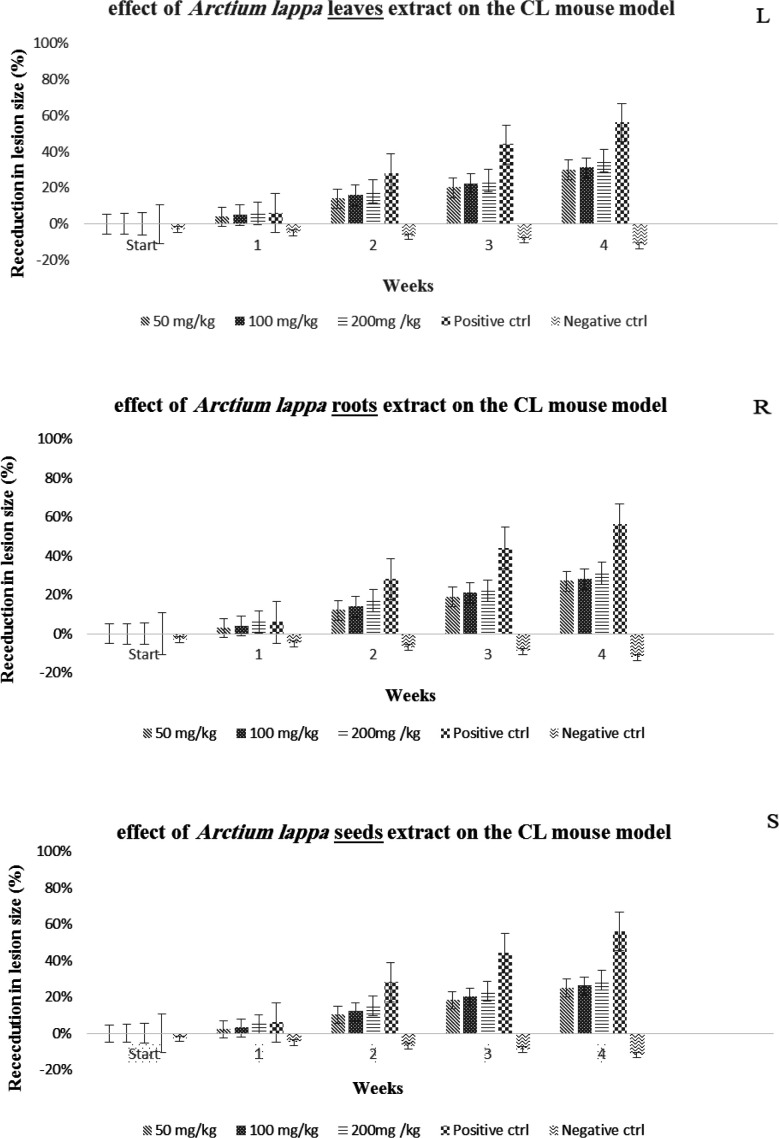
percentage of wound reduction in the groups treated with hydroalcoholic extracts of leaves, roots, and seeds of the *Arctium* *lappa*. L,leaves; R, roots; S,seeds. By using the two-way ANOVA statistical method, the significant effect of the increase in concentration (L: p≤0.001, R: p=0.02, S: p=0.03), the increase in exposure time to the extract (L: p≤0.001, R: p≤0.001, S: p≤0.01) and the interaction effect of these two factors (L: p=0.002, R: p≤0.001, S: p≤0.001) on reducing the size of the wound was determined. According to the obtained results and the comparison of the average lesions, it could be suggested that greatest effect is related to Glucantime, then the concentration of 200 mg/kg of leaves, roots and seeds respectively. Also, the statistical analysis of Variance test showed that in each group of L, R and S extracts, the effect of the extract increases with the increase of concentration and exposure time (p≤0.001). Positive control (Positive ctrl), Negative control (Negative ctrl).

**Figure 5:**
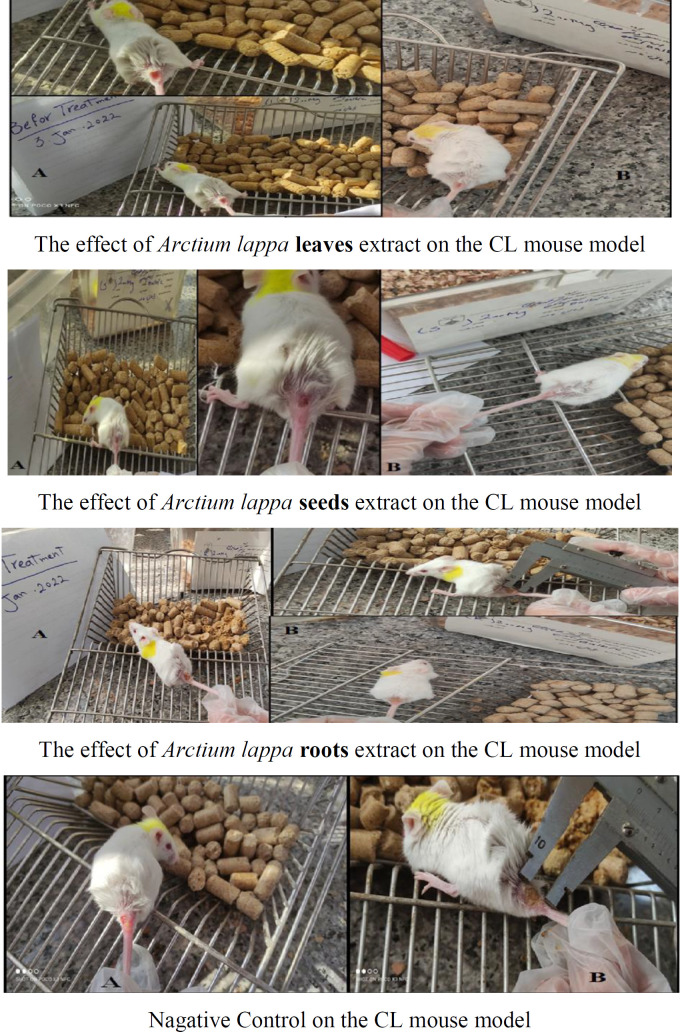
The effect of *Arctium lappa *leaves, roots and seeds extract (the concentration of 200 mg/kg) on the CL mouse model before (A (and after (B (treatment compared to the negative control. CL, Cutaneous Leishmaniasis.

The results of this research are shown in Figures 1-5 and Tables 1 and 2. From the results of the effects of the extracts on the promastigote form of the parasite by two counting and MTT methods, due to the almost identical results, the viability percentage of the promastigote by the MTT method is presented in Table 1 and the analysis of this results shows the effect of concentration (L: p≤0.001 (S: p≤0.001 R: p≤0.001), exposure time (L: p=0.002 S: p≤0.001 R: p=0.004) and the interaction effect of both factors (L: p≤0.001 S: p≤0.001 R: p≤0.001) is significant on reducing the number of promastigotes. Also, by performing Tukey's post hoc test, it was found that there is a significant relationship between the different concentrations of the hydroalcoholic extract of the leaves, roots and seeds of the burdock plant with each other and the negative control. There was no significant relationship between the concentrations of 50, 25, and 12.5 µg/ml of the leaves, roots, and seeds with the positive control (Glucantime) (p>0.05) whereas a significant relationship was observed between the higher concentrations of 100, 200, and 400 µg/ml with the positive control (p≤0.05). On the other hand, amphotericin B with high lethality and very low percentage of live parasites exhibited a significant difference across the concentration gradient (p≤0.05). 

The results of cytotoxicity of different concentrations of* Arctium* *lappa* on living macrophage cells are shown in Figure 2. Two-way ANOVA statistical test showed the significant effect of the concentration of the extracts (L: p≤0.001, R: p≤0.001, S: p≤0.001), the exposure time (L: p≤0.001, R: p=0.004, S: p≤0.001) and the interaction effect of these two factors (L: p=0.002, R: p=0.019, S: p=0.024) on the viability of macrophages. Also the significant relationship was found between the different concentrations of the hydroalcoholic extract of the leaves, roots and seeds of the *Arctium* *lappa* with each other and with the negative control (p≤0.05).

According to the results shown in Table 1, the highest inhibitory effect of the prepared extracts on *L.majar* amastigote was in the order of leaves (IC50_ 24hr_ =155 and IC50_ 48hr_ =25 µg/ml) then the roots (IC50_ 24hr_ =210 and IC50_ 48hr_ =50 µg/ml) and finally the seeds (IC50_ 24hr_ = 470 and IC50_ 48hr_ =181 µg/ml). Regarding the results of the promastigote form of the parasite, the highest inhibitory effect was related to the seeds (IC50_ 24hr_ = 470 and IC50_ 48hr_ = 420µg/ml) then the roots (IC50_ 24hr_ =983 and IC50_ 48hr_ =771 µg/ml) and finally the leaves extract of *Arctium lappa* (IC50_ 24hr_ = 1025 and IC50_ 48hr_ = 920 µg/ml).

The viability % of the intracellular form of the parasite, which was evaluated by the amastigote-macrophage model, and the significant effect of extract concentration, treatment time and the interaction effect of these two factors on the intracellular form of the parasite are shown in Figure 3.

The results of the *in vivo* phase and the effect of the hydroalcoholic extract of the leaves, roots, and seeds of the *Arctium* *lappa* on wound healing caused by *Leishmania major* in BALB/c laboratory mice are shown in Figure 4. The analysis of the results of this step determined the effect of concentration (L: p≤0.001 S: p=0.03 R: p=0.02), exposure time (L: p≤0.001 S: p≤0.01 R: p≤0.001) and the interaction effect of concentration and exposure time (L: p=0.002 S: p≤0.001 R: p≤0.001) is significant and showed a greater decrease in lesion size in the groups treated with higher concentrations and in the third and fourth weeks. It was also found out there is no significant difference between the first and second week after starting the treatment (p>0.05), but at the end of the third and fourth week, this difference was significant (p≤0.05). The results are shown in Figure 4.

The effect of extracts of leaves, roots and seeds of Arctium lappa in mouse model of Coutaneous Leishmaniasis (CL) before and after treatment and negative control is shown in Figures 5. The selectivity index (SI) of each extract of the leaves, roots and seeds of *Arctium lappa*, which is defined based on the ratio of the toxic concentration of a sample to its effective bioactive concentration (CC_50_/IC_50_), is shown in Table 1. The results showed that the values of SI for all concentrations of three types of extracts were greater than one.

The comparison of the lesion size reduction (%) and the spleen and liver parasite burden in CL mouse model in the groups treated with extracts is summarized in Table 2.

## Discussion

Today, leishmaniasis is not only a disease, but in a broader and more realistic view, it is considered a very important and challenging health problem in a wide range of human societies (Tiuman et al., 2011). At present, pentavalent antimony compounds including sodium stibogluconate (Pentostam) and meglumine antimony (Glucantime) are the most important drugs of choice used in the treatment of leishmaniasis (Brito et al., 2017). Research has shown that these drugs have disadvantages such as toxicity, side effects, recurrence rate and high cost, long treatment period and the resistance that the parasite has recently shown to these drugs, which have prompted researchers to look for medicinal compounds with more economical and effective characteristics, with fewer side effects (Milani et al., 2011). Since herbal medicines are suggested to present less harmful side effects, and are more readily accessible than chemical medicines, as a result, it can be said that the use of native plants of any region can be a source of medicinal agents, including anti-leishmania, due to their effective compounds. One of these plants is *Arctium lappa* and many researches point to its medicinal properties such as antibacterial, antiviral, antifungal, anti-allergic, immune system strengthening, anti-tumor, anti-inflammatory, sugar-reducing and blood pressure reducing effects. (Janaćković et al., 2004; Rocha et al., 2005). *Arctium lappa *contains effective biological compounds such as caffeic acid, rutin, cynarin, quercetin, luteolin, diarchetigenin and arctigenin. The compounds in the plant seeds include chlorgenic acid, cynarin, arctin, arctigenin, etc. The seed consists of linoleic acid and oleic acid, and these fatty acids have anti-inflammatory and antioxidant properties (Almoradie et al., 2018). The compounds found in the roots of the plant include chlorgenic acid, inulin, cynarin, quercetin, arctin and luteolin (Wang et al., 2019). In the study conducted by Hanoon et al. the effect of aqueous and alcoholic extract of *Arctium lappa* has been investigated only on the promastigote form and it was shown that the concentration of 1.5 mg/ml had the greatest effect, whereas the hydroalcoholic extract had a better inhibitory effect than the aqueous extract (Hanoon et al., 2022). In the study of Farghi Yamchi et al., where the methanol extract of the root was investigating, IC_50_ = 131.25 µg/ml after 24 hr was reported, which was higher in our study. Also the average number of amastigotes in macrophages after 24 hr at concentrations of 500 and 1000 μg/ml was reported 3.52 and 2.02, respectively, and in our study with a lower concentration (400 μg/ml) it was 4.5. (Farghi Yamchi et al., 2016). The results of our study also confirm the anti-leishmanial effect of this plant. The growth inhibition of promastigotes in the presence of different concentrations of hydroalcoholic extracts of roots, seeds and leaves after 24 and 48 hours compared to the negative control was significant and dependent on the concentration and exposure time (p≤0.001). The results showed that among the investigated extracts, the seed extract (IC_50_ = 480 and 420 µg/ml) had a greater effect on inhibiting promastigote growth than the roots (IC_50_ = 983 and 771 µg/ml) and leaves (IC_50_ = 1025 and 920 µg/ml), and consequently the IC_50_ value reported in the time intervals of 24 and 48 hr was the lowest for the seed extract and the highest for the leaves extract (Figure 1). If in the amastigote stage, the greatest effect was related to the leaves extract (IC_50_ = 155and 25 µg/ml), then the roots (IC_50_ = 210 and 50 µg/ml) and finally the seeds (IC_50_ = 470 and 181 µg/ml) (Figure 3). 

On the other hand, the results of the effect of the hydroalcoholic extract of the leaves, roots and seeds of the *Arctium lappa* plant in different concentrations on the viability of macrophages (cell line J774 after 24 and 48 hr) show that the highest CC_50_ value was related to the roots then the seeds and finally the leaves (Figure 2). Although by calculating the SI index (the ratio of CC_50_ to IC_50_) for all the extracts, it was found that the prepared extracts did not have significant toxicity, but based on these results, the toxicity of the leaves and seeds was lower than that of the roots. These results are consistent with studies such as Dias and colleagues who investigated the anti-schistosomal and antiviral activities of *Arctium lappa* against Schistosoma mansoni and herpes simplex virus-1 in laboratory conditions and showed that *Arctium lappa* in high concentrations such as 400 μg/ml had no cytotoxic effect on Vero cells (Dias et al., 2017; Koriem et al., 2016). In the in vivo phase of this study, the effect of three concentrations of 50, 100, and 200 mg/kg hydroalcoholic extracts of the leaves, roots, and seeds of *Arctium lappa* on the wounds caused by Leishmania major in BALB/c mice was investigated for a period of 4 weeks. The findings regarding the examination of the wound diameter in the treatment groups under study show that in the fourth week after the challenge, the average diameter of the wounds in all groups has decreased compared to the negative control group. Also, the results showed that the highest percentage of wounds diameter reduction at the end of the treatment period in different treatment groups compared to the control group was related to Glucantime, followed by leaves, roots and seeds extracts. In addition, there was a significant difference between similar concentrations of the extracts together, and between different concentrations (p≤0.05). The concentration of 200 mg/kg of each type of extract had a greater effect than other analyzed concentrations. In fact, the results show that in each group of the desired extracts, the effect of the desired extract increases with the increase in concentration and the passage of time (p≤0.001). In terms of the status of the parasite load in the spleen of the mice under study, the greatest decrease in the parasite load was related to the hydroalcoholic extract of the leaves compared to other groups. It seems that the anti-leishmanial effect of this plant is related to the compounds present in the different organs of the plant, including the biologically active lignans arctigenin (a dietary phytoestrogen) and its glycoside, arctin (lignanolides) which are usually present in the seeds, roots, fruits and leaves of *Arctium lappa *(Wang et al., 2019). Arctigenin extracted from *Arctium lappa* shows interesting activity due to its ability to reduce the levels of nitric oxide (NO) and pro-inflammatory cytokines (Knipping et al., 2008; Pospíšil et al., 2021). Also, due to the presence of beta-sitosterol (β-sitosterol) in this plant and its effect in the treatment of leishmaniasis lesions caused by L.tropica compared to ketoconazole and mupirocin in other studies, the healing effect of the lesion can be attributed to the presence of this substance (Pramanik et al., 2020).

Among the polyphenols of this plant, caffeic acid has anti-promastigote activity with an apoptosis-like process and anti-amastigote activity with the production of TNF-α/ROS/NO and reducing the availability of iron. Studies have shown that caffeic acid, in addition to increasing NO production by macrophages infected with Leishmania parasites, can inhibit arginase and improve visceral leishmaniasis (Qian et al., 2016; Bortoleti et al., 2019; Alson et al., 2018).

Another polyphenol of this plant is chlorogenic acid, studies have shown that this compound inhibits the cell cycle of *Leishmania donovani* and modulates cytokines and nitric oxide in laboratory conditions thereby exerting potential anti-leishmanial effects (Majumder et al., 2020; Manjolin et al., 2013). Luteolin is another polyphenol isolated from burdock plant that inhibits arginase, the central enzyme in Leishmania (*Leishmania amazonensis*) infection. This non-toxic polyphenol seems to be a strong candidate for anti-leishmanial drug design. (Majumder et al., 2020; Manjolin et al., 2013). Also, quercetin is one of the other polyphenols isolated from this plant, which inhibits the proliferation and migration of fibroblasts, whilst also playing a role in the inhibition of inflammation and increase the expression of growth factors in mice through Wnt/β-catenin and telomerase reverse signaling pathways via transcriptase (TERT) and thus promote wound healing (Abdeyazdan et al., 2022). This study showed the anti-leishmanial effects of the ethanol extract of Arctium lappa leaves, roots and seeds *in vivo* and *in vitro* conditions. Due to its diverse and effective compounds, this plant has the capability of more specific research to separate its fractions and investigate their anti-leishmanial effects in order to find an effective herbal compound for the treatment of cutaneous leishmaniasis.

**Table 1:** IC50 and CC50 values and SI indices of the three extracts of leaves, roots and seeds of *Arctium* *lappa*

**Compound**			**Time** **(hour) **	**Anti-promastigote activity IC50 (µg/ml)**	**Anti-amastigote activity IC50 (µg/ml)**	**Toxicity** **CC50(µg/ml)**	**Selectivity index** **(SI)**
Seeds			24	480±3.5	470±7.07	1800±12.8	3.8±2.5
			48	420±1.2	181±8.84	1550±13.94	8.5±0.3
Leaves			24 48	1025±3.78 920±9.3	155±4.82 25±7.80	2000±10 1600±15.2	12.9±2.92 64±1.87
Roots			24	983±5.96	210±3.62	1700±14.3	8±1.9
Glucontime (ctrl pos) Amphotericine B			48 24 48 24 48	771±9.35 1720±6.87 1660±7.87 0.06	50±3.2 349±2.78 53±4.78	1500±18.2 2550±14.5 2040±22.2	30±2.2 8.5±0.5 24±1.4

**Table 2:** Percentage of lesion size reduction and parasite burden among the different groups of CLmouse model exposed to different concentrations of extracts (leaves, roots and seeds of *Arctium* *lappa*).

**Type of extract**	**Concentrations (µg/ml)**	**Reduction in Lesion size (%)**	**Spleen parasite burden (LU)**	**Liver parasite burden (LU)**
	50	25%	260±3.5	252±4
Seeds	100	26%	245±2.78	245±2.9
	200	29%	230±4.2	220±3.48
Leaves	50 100 200	30% 31% 35%	195±5 190±1.72 185±2.28	182±2.42 180±3.42 170±5.2
Roots	50 100 200	27% 28% 31%	235±2.25 220±6.78 195±3	210±3 190±8.92 180±2.78

**CL, **Cutaneous Leishmaniasis. Note: The highest reduction in lesion size (%) and parasite burden in CL mouse model was respectively related to groups treated with Glucantime, extracts of leaves, roots and finally seeds of *Arctium lappa*. (in Table each point represents the mean±SD of at least three experiments).
